# Aberrant IgA1 Glycosylation in IgA Nephropathy: A Systematic Review

**DOI:** 10.1371/journal.pone.0166700

**Published:** 2016-11-21

**Authors:** Qiang Sun, Zhenhai Zhang, Hong Zhang, Xiaorong Liu

**Affiliations:** 1 Beijing Children’s Hospital affiliated to Capital Medical University, Nephrology Department, Beijing Key Laboratory of Pediatric Chronic Kidney Disease and Blood Purification, Beijing, 100045, China; 2 The Affiliated Bayi Brain Hospital, The Military General Hospital of Beijing PLA, Beijing, 100053, China; University Jean MONNET of SAINT-ETIENNE, UNITED STATES

## Abstract

**Objective:**

Galactose-deficient IgA1 was evaluated in patients with IgA nephropathy(IgAN) and controls in order to determine the predictive value of galactose-deficient IgA1 in cases of IgA nephropathy.

**Methods:**

PubMed, EMBASE, Cochrane central register of controlled trials, CNKI, CBM disc, and VIP database were searched to identify eligible studies that evaluated a difference in aberrant IgA1 glycosylation in IgAN patients compared with controls. A meta-analysis was conducted to evaluate the impact of galactose-deficient IgA1(Gd-IgA1) levels in different groups.

**Results:**

A total of 22 studies (n = 1657) met inclusion criteria. The mean Newcastle-Ottawa Scale (NOS) score was 7.2 and ranged from 6 to 8. The standard mean difference(SMD) in the meta-analysis of 20 studies of the level of Gd-IgA1 in the serum and/or supernatant of cultured cells was higher in the IgAN group compared with healthy controls as well as in those with other renal diseases (SMD = 1.76, 95% CI = 1.18–2.34, P<0.00001; SMD = 1.05, 95% CI = 0.05–2.04, P = 0.04). The data synthesis suggested that IgAN patients had similar levels of serum Gd-IgA1, with no significant differences, compared with first-degree relatives and Henoch-Schonlein purpura nephritis (HSPN) patients (MD = 0.04, 95% CI = 0.00–0.08, P = 0.05; MD = -46.03, 95% CI = -217.70–125.64, P = 0.60). In addition, the combined MD of 5 studies indicated that there were no significant differences in Gd-IgA1 levels among patients with varying severities of IgAN (MD = 0.02, 95% CI = -0.02–0.05, P = 0.28).

**Conclusions:**

The pooled evidence suggests that the level of Gd-IgA1 in the serum or supernatant of cultured cells from peripheral blood or tonsils may be a useful biomarker for predicting IgA nephropathy, though the level of Gd-IgA1 was not significantly associated with disease severity.

## Introduction

IgA nephropathy (IgAN) is a common primary glomerular disease. It had previously been believed to be a benign illness, but is currently considered to be a progressive disease characterized by a gradually decreasing glomerular filtration rate (GFR), which results in end-stage renal disease (ESRD) in 15% to 20% of patients within 10 years and in 30% to 40% of patients within 20 years of disease onset[1,2].

Thus far, the gold standard for the diagnosis of IgAN has been pathological analysis of the kidney tissue following renal biopsy. However, some patients refuse to have this done since it is an invasive, and often traumatic, procedure. In addition, there can be complications with biopsy procedures, such as hemorrhage, perirenal hematoma, and arteriovenous fistula. Therefore, an alternative, non-invasive method for diagnosing IgAN would be very beneficial.

IgAN appears to be a systemic disease. The pathogenesis of IgAN is not clear, though it is accepted that an aberrant glycosylation pattern of IgA is involved. In IgAN, the mesangial deposits of IgA contain high concentrations of abnormally O-glycosylated IgA1, characterized by undergalactosylation[3]. Some studies have suggested that variants of galactose-deficient IgA1(Gd-IgA1) are more common in the sera of IgAN patients compared with the sera of healthy individuals or with sera from patients with other types of renal disease[4,5]. Furthermore, some researchers have reported that the level of Gd-IgA1 in the sera of patients with IgAN is associated with disease progression[6], though others have found that serum Gd-IgA1 level is not associated with proteinuria in children with IgAN[7]. In the current study, a meta-analysis was done to determine differences in Gd-IgA1 serum levels between IgAN patients and healthy controls and to clarify whether serum assays for Gd-IgA1 are reliable and useful for predicting renal pathological progression of IgAN.

## Methods

### Identification and Selection of Studies

This systematic review was performed according to the Cochrane Handbook for Systematic Reviews of Interventions and the Preferred Reporting Items for Systematic Reviews and Meta-Analyses (PRISMA)[8]. (S1 File)

Eligible studies were included if all criteria were met as follows: (1) studies were case controlled or cohort studies; (2) patients in one group were diagnosed with primary IgAN via a renal biopsy showing IgA as the dominant or co-dominant Ig in a typical mesangial distribution, in the absence of clinical and laboratory evidence for systemic disease[9]; (3) patients in the control group were healthy controls from community, first-degree relatives of patients with IgAN, or patients with diseases other than IgAN; (4) the study analyzed samples from sera or the supernatant of cultured cells from subjects; (5) Gd-IgA1 levels were determined by ELISA. In previous studies, O-glycans in the hinge region of IgA1 have been determined by ELISA using lectin-specific binding.

Six databases (PubMed, EMBASE, Cochrane central register of controlled trials, CNKI, CBM disc, vip) were searched on January 1st, 2015. The evaluable studies, systematic reviews, meta-analyses, and reports were manually searched for additional studies that met inclusion criteria. No restriction on language or publication status was applied. The search terms for IgAN included Glomerulonephritides, IGA and Berger's Disease and Berger’s Disease and IGA Glomerulonephritis and IGA Nephropathy and Immunoglobulin A Nephropathy and Nephropathy, Immunoglobulin A and Nephritis, IGA Type and IGA Type Nephritis and Nephropathy, IGA and Berger Disease and IgA Nephropathy 1 and Nephropathy 1, IgA. The search terms for glycosylation were glycosylations and Protein Glycosylation and Glycosylation, Protein. The detailed search strategy is shown in S1 Fig.

### Data extraction

**Fig 1:**
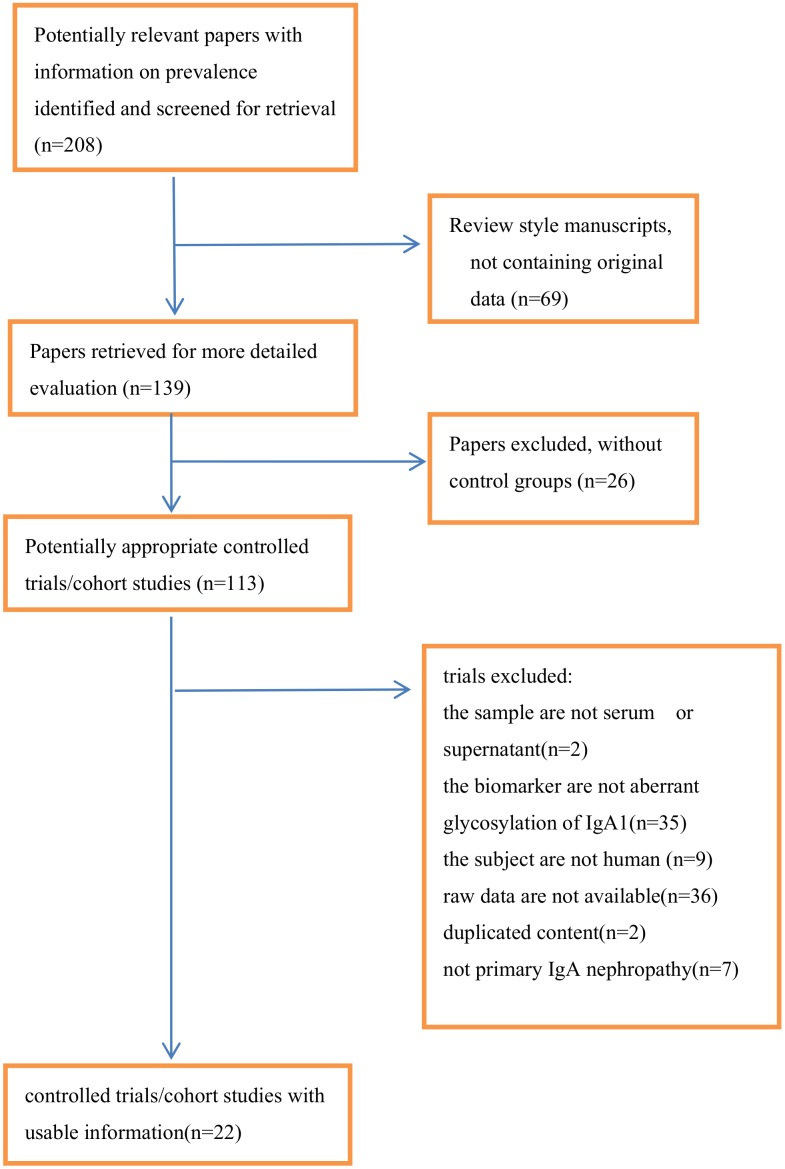
Flow chart of the meta-analysis.

Two reviewers independently screened the initially identified studies. Full-text articles of potentially eligible studies were independently assessed against the eligibility criteria. Differences were then compared and referred to consultants for resolution. When more than one paper was derived from the same study, only the most potentially eligible records were included, considering the integrity and availability of the data. The main reasons for exclusion of trials are described in Fig 1.

The same two reviewers independently extracted the data from each study. The following variables pertaining to patients and setting were collected: publication year, number of participating centers, total number of patients, groups, clinical setting, protocol for testing method (sample, agents, method, dose, company), and major outcomes. Differences were then compared and referred to consultants for resolution.

### Risk of Bias

Per the Cochrane Collaboration, the Newcastle-Ottawa Scale (NOS) was used to assess the quality of included studies via judging them using three board perspectives: the selection of study groups, the comparability of study groups, and the measurement of exposure in study groups[10].

### Data synthesis and statistical analysis

The meta-analysis was conducted using Review Manager 5.3. Heterogeneity arising from factors and other threshold effects were analyzed by Chi-square and Cochran-Q. The inconsistency index (I-squared) was computed as a measure to quantify the amount of heterogeneity. An I^2^ of 25% to 49% was considered to be low, an I^2^ of 50% to 74% was considered to be moderate, and an I^2^> 75% was considered to be a high degree of heterogeneity. The Random-effect model was used to pool several indexes. When there was high heterogeneity, subgroups and sensitivity analyses were used to examine the potential source of heterogeneity. If there was consistent heterogeneity among individual studies, the standard mean difference (SMD) was used instead of the mean difference (MD) given the clinical importance of some indexes. A pooled MD>0 indicated more difference between the two groups, and was considered to be significant if the 95%CI did not overlap 0, with p<0.05.

## Results

### Study Selection and Characteristics

A total of 208 papers were identified. Of these, only 22 were eligible for inclusion in the analysis (see flow chat) (Table 1). Fifteen of the 22 were written in English and 7 were in Chinese. The 22 trials that were analyzed included 1657 participants (301 children included). Of the 22 included trials, Gd-IgA1 was detected by biotinylated N-acetylgalactosamine(GalNAc)-specific lectin from Helix aspersa (HAA) in 8 and by biotinylated Vicia villosa lectin binding assay (VVL) in 14. In studies using HAA, 2 trials expressed the results in units/ml, where 1 unit of Gd-IgA1 was defined as 1ug of the standard Gd-IgA1 protein. The other 6 trials described the HAA–IgA1 binding level in each sample as a relative value calculated by the absorbance level divided by the absorbance level of the quality control in the same plate. The color reaction was stopped with 1 M sulphuric acid and absorbance at 490 nm was measured. In studies using VVL, 8 of the 14 analyzed serum samples and 6 analyzed supernatants from cultured cells. Three of the 14 trials expressed the results as AU/ml or AU and the remainder as relative ratio.

**Table 1:** Data of the included 22 studies.

Study	Year	Country	Study Population	Mean age(yr)	Male (%)	Sample	Detection Index
Zou MS[11]	2012	CN	15 IgAN with 15 HSPN with 15 HC	8.2; 8.4; 9.5	8(53);9(60%);7(47%)	serum	HAA-IgA1
Kenji S[12]	2014	JPN	32 IgAN with 20 HC	30.3; 35.1	11 (34%); 12 (60%)	serum	HAA-IgA1
Krzysztof K[13]	2011	USA, JPN	14IgAN;20HSPN;51Pediatric Controls;25Relatives of IgAN;29Relatives of HSPN;141Adult Controls	14.3;10.1;15.7; 43.0;39.2;36.6	10(71%);13(65%);28(55%); 11(44%);12(41%);73(52%)	serum	HAA-IgA1
KS B[14]	2008	UK	12 IgAN with 13 matched controls undergoing elective orthopedic surgery	39; 37	10 (83%); 7 (54%)	serum	HAA-IgA1
Sachiko S[15]	2008	JPN	41IgAN; 43other kidney disease;38 HC	32.7;53.4;31.0	20(49%);27(63%);16(42%)	serum	HAA-IgA
Linossier [16]	2002	FR	44IgAN: 22normal GBM; 22 thin GBM; 22 HC	42;41;NA	22(50%);NA	serum	HAA-IgA1
Lin[17]	2009	CN	63IgAN;32 first-degree relatives;44 spouses of 44 patients;39HC; 26Hass I–III; 37 Haas IV–V	33.7; 37; 35.8; NA	32(50.8%);16(50%);19(43.2%);NA	serum	HAA-IgA1
Berthoux [18]	2012	FRA	97IgAN;30HC;30other disease (15 membranous nephropathy and 15 with biopsy-proven nephro-arteriolosclerosis) IgAN:ARR = 0;ARR = 1;ARR = 2;ARR = 3	43.6; 45.7; 37.0	73 (75%); 20 (66.7%); 20 (66.7%)	serum	HAA-IgA1
Jiang XY[19]	2009	CN	26 IgAN with 20 HC IgAN: 10 hemoturia with 6 hemoturia+proteinuria with 10 NS;HC:20 IgAN: 5I+II;11III;10IV+V*;HC:20	9; NA	21 (81%); 16 (80%)	serum	VVL-IgA1
Zhang J[20]	2007	CN	15 IgAN with 10 HC	33.2;30.0	3(20%);4(40%)	supernatant of cultured B lymphocyte from peripheral blood	VVL-IgA1
Yan Y[21]	2006	CN	10mmpIgAN;10FpsIgAN;10HC	NA	NA	serum	VVL-IgA1
Fu SX[22]	2003	CN	68IgAN;20MCD	30;23	39(57%);11(55%)	serum	VVL-IgA1
He LY[23]	2013	CN	22IgAN;24ct	33.2;19.2	13(59%);13(54%)	supernatant of cultured mononuclear cells from tonsil	VVL-IgA1
Jing SH[24]	2014	CN	11IgAN;11ct+sas	30.8;15.7	6(55%);7(64%)	supernatant of cultured mononuclear cells from tonsil	VVL-IgA1
Ling J[25]	2014	CN	21 IgAN with 10 HC	27.5; 29.5	11 (52%); 6 (60%)	supernatant of cultured B lymphocyte from peripheral blood	VVL-IgA1
Linshen X[26]	2013	CN	18 IgAN with 12 HC	32.2; 29.5	10 (56%); 7 (58%)	supernatant of cultured B lymphocyte from peripheral blood	VVL-IgA1
J.-X. D[27]	2008	CN	70 IgAN with20 HC	30.8; NA	41 (59%); NA	serum	VVL-IgA1
L.-X. X[28]	2005	CN	20 mmpIgAN;20 Fps IgAN;20 HC	29.7;34.7;NA	NA	serum	VVL-IgA1
Alice C[29]	1999	UK	22IgAN; 23 HC	43.5; 43.0	14 (64%); 15 (65%)	serum	VVL-IgA1
Alice C[30]	1998	UK	24HSPN;22HSP;7post-streptococcal glomerulonephritis;22HC	7.0;5.5;7.5;7.0	14(58%);10(45%);5(71%);10(45%)	serum	VVL-IgA1
			31HSPN;9IgAN;11mesangial proliferative glomerulonephritis;22HC	37.5;32;49;36	17(55%);2(22%);6(55%);13(46%)		
A. C. Allen[31]	1997	UK	9IgAN; 12 HC	33;38	6(67%);6(50%)	serum	VVL-IgA
Sun[32]	2015	CN	26IgAN; 11other renal disease; 13HC	9.93;8.80;12.12	19(74.1%);8(72.7%);9(69.2%)	supernatant of cultured B lymphocyte from peripheral blood	VVL-IgA1

IgAN,IgA nephropathy patient;HC,healthy control;NS, nephrotic syndrome;Gd-IgA1,Galactose—Deficient IgA1; NA, not answer; PGD,primary glomerula disease; MCD,minimal change nephrotic syndrome; LN,lupus nephritis;LPS,lipopolysaccharide;AMI,Astragalus membranaceus injection; HSPN,Henoch-Schönlein purpura nephritis;GBM,glomerular basement membrane; ARR, absolute renal risk; ct, chronic tonsillitis; sas, and sleep apnea syndrome; mmp, mild mesangial proliferative; Fps, Focalproliferative sclerosis

*graded by 1982 WHO

### Quality Assessment of Included Studies

The qualities of included studies, assessed with NOS(Newcastle-Ottawa Scale), are provided in Table 2. The mean total score was 7.2 with a range from 6 to 8. Three of the 22 studies are of less good quality and scored 6, while the other 18 studies are of good quality and scored 7–8.

**Table 2:** Quality assessment of the 22 included studies with the Newcastle-Ottawa Scale (NOS).

Reference	year	Selection of subjects/4	Comparability of groups/2	Measurement of Exposure/4	Total score of NOS/10
Zou MS[11]	2012	3	2	2	7
Kenji S[12]	2014	3	1	2	6
Krzysztof K[13]	2011	3	2	2	7
KS B[14]	2008	3	2	2	7
Sachiko S[15]	2008	3	2	2	7
Linossier [16]	2002	3	1	2	6
Lin[17]	2009	4	1	2	7
Berthoux [18]	2012	3	2	2	7
Jiang XY[19]	2009	4	1	2	8
Zhang J[20]	2007	4	2	2	8
Yan Y[21]	2006	3	1	2	7
Fu SX[22]	2003	3	1	2	6
He LY[23]	2013	3	2	2	7
Jing SH[24]	2014	3	2	2	7
Ling J[25]	2014	4	2	2	8
Linshen X[26]	2013	4	2	2	8
J.-X. D[27]	2008	4	1	2	7
L.-X. X[28]	2005	4	1	2	7
Alice C[29]	1999	4	2	2	8
Alice C[30]	1998	4	2	2	8
A. C. Allen[31]	1997	4	2	2	8
Sun[32]	2015	4	2	2	8

### IgAN versus healthy controls

A total of 974 samples, from 20 studies, were included. Patients with IgAN had higher levels of Gd-IgA1 in serum and in the supernatant of cultured cells compared to healthy controls (P<0.00001, Heterogeneity: I^2^>70%). Subgroups were then established based on the method of Gd-IgA1 detection: HAA and VVL subgroups. (S2 Fig).

**Fig 2:**
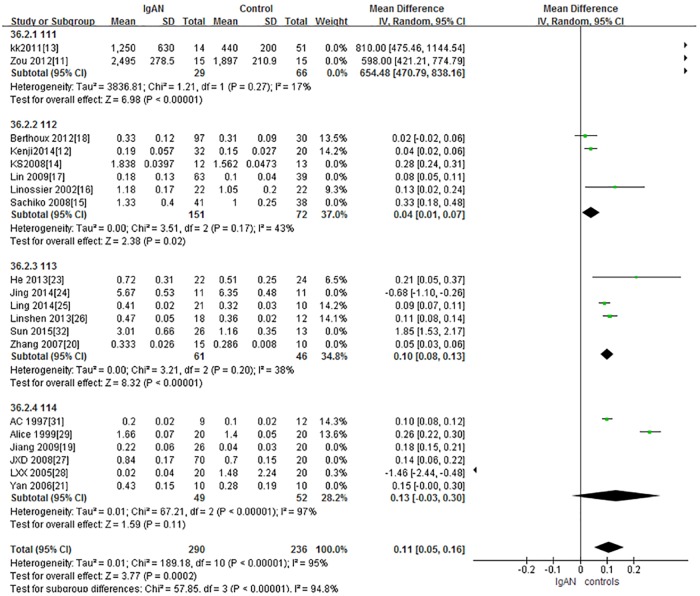
The forest plots of the comparation between IgAN group and control group.

A total of 8 studies, containing 524 samples, were included in the HAA lectin subgroup. In accordance with the above results, IgAN patients in the HAA subgroup had significantly higher levels of Gd-IgA1 compared with healthy controls (P<0.0001, Heterogeneity: I^2^ = 89%). Further subgroups were then established and the results were similar (Fig 2).

In HAA lectin subgroup, there were 2 studies of children in which 95 participants were included and 6 studies of adults in which 429 participants) were included (S2-111 and S2-112 Fig). Children with IgAN had significantly higher levels of Gd-IgA1 compared with healthy children (P<0.00001, Heterogeneity: I^2^ = 17%),(Fig 2-111). Adults with IgAN also had significantly higher levels of Gd-IgA1 compared with healthy controls (P = 0.001, Heterogeneity: I^2^>70%),(S2-112 Fig). A sensitivity analysis was done since I^2^>70%. When 2 trials were excluded, the heterogeneity decreased (I^2^<70%), as well as when 1 trial was excluded (I^2^<50%) and the results remained the same, P<0.05.(Fig 2-112)

In VVL lectin subgroup, there were 12 studies in which 450 participants were included. It showed higher level of in IgAN patients had significantly higher levels of Gd-IgA1 compared with healthy controls (P<0.0001, Heterogeneity: I^2^>70%). (S2 Fig).

The VVL lectin group was divided into 2 subgroups. One included 6 studies, with 257 participants, in which serum was used for Gd-IgA1 detection and the other included 6 trials, with 193 participants, in which supernatant was used for Gd-IgA1 detection. The level of Gd-IgA1 was significantly higher in IgAN patients compared with controls in both the serum (P = 0.01, Heterogeneity: I^2^>70%), (S2-114 Fig) and supernatant (P = 0.01) subgroups(S2-113 Fig). Since I^2^>70 in the serum subgroup, a sensitivity analysis was performed. I^2^ = 0% after 3 trials were excluded and the results remained the same (P<0.00001).(Fig 2-114). A sensitivity analysis was also performed in the supernatant subgroup to keep heterogeneity. I^2^<40% after 3 trials were excluded and the results remained (P<0.00001). (Fig 2-113)

### IgAN versus relatives

**Fig 3:**
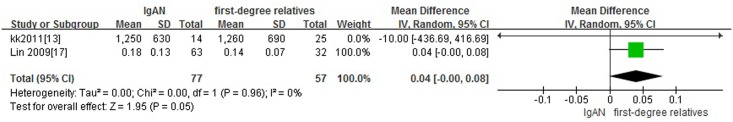
The forest plots of IgAN group and first-degree relatives group.

Two studies with 134 adults participants were included. IgAN patients had similar levels of serum Gd-IgA1 compared to their first-degree relatives (P = 0.05, Heterogeneity: I^2^ = 0%) (Fig 3).

### IgAN versus HSPN

Two studies with 64 children participants were included. There was no significant difference in Gd-IgA1 levels between children with IgAN and children with HSPN (P = 0.60. Heterogeneity: I^2^ = 0%).

**Fig 4:**
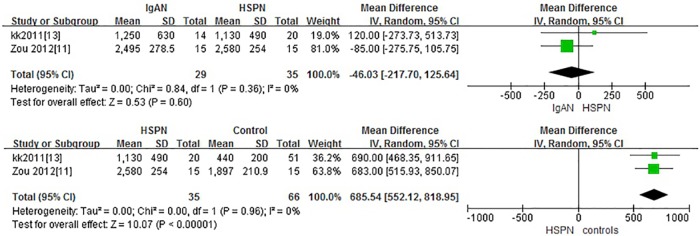
The forest plots of the comparation among IgAN group, HSPN group and controls.

Three studies with 101 adults and children participants were included. Patients with HSPN had significantly higher levels of Gd-IgA1 compared to healthy controls (P<0.00001, Heterogeneity: I^2^ = 82%). Sensitivity analysis: I^2^ = 0% after 1 trial was excluded and only children were included and the results remained the same. (P<0.00001) (Fig 4).

### IgAN versus other renal disease

**Fig 5:**
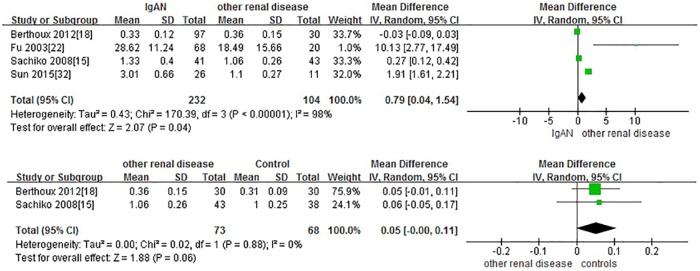
The forest plots of IgAN group and other renal disease group.

Four studies with 477 adults participants were included. IgAN patients had significantly higher levels of Gd-IgA1 compared to patients with other renal diseases(minimal change disease (MCD), membranous nephropathy, biopsy-proven nephro-arteriolosclerosis, Alport syndrome, thin basement nephropathy and mesangial proliferative glomerulonephritis) (P = 0.04, Heterogeneity: I^2^ = 93%). The Standard Mean Difference (SMD) was used. There is no significant difference between Gd-IgA1 levels in patients with other renal diseases group and healthy controls. (P = 0.06, Heterogeneity: I^2^ = 0%) (Fig 5).

### IgAN and severity

**Fig 6:**
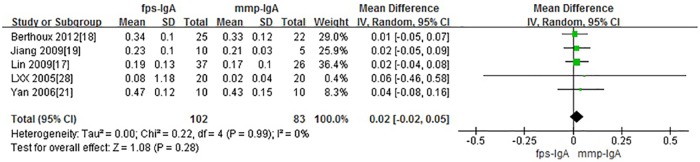
The forest plots of comparison among variable grades of IgAN severity.

A total of 5 studies with 185 participants were included when comparing Gd-IgA1 levels in cases of severe IgAN versus mild IgAN. Four comparisons were based on histopathologic grading: mild mesangial proliferative IgAN (mmp) versus focal proliferative sclerosis IgAN (fps); I+II versus IV+V; I-III versus IV-V(graded by 1982 WHO). One was based on absolute renal risk (ARR): 1 versus 3. There is no significant difference in serum Gd-IgA1 levels in patients with severe IgAN compared to those with mild IgAN. (P = 0.25, Heterogeneity: I^2^ = 0%) (Fig 6)

## Discussion

In the current meta-analysis, both adults and children with IgAN had significantly higher serum levels of Gd-IgA1 compared with healthy controls. In addition, the level of Gd-IgA1 in the supernatant of cultured B lymphocytes form peripheral blood or mononuclear cells from the tonsils of IgAN patients was significantly higher compared to healthy controls. This indicates that serum Gd-IgA1 may be an important and useful biomarker for the diagnosis of IgAN. Testing serum Gd-IgA1 would not be used to replace renal biopsy as the gold standard for diagnosis. Rather, in clinical practice, serum Gd-IgA1 could be tested initially in cases where a patient or parent of a child that is a patient is hesitant about doing the biopsy due to potential risks. In patients with highly elevated Gd-IgA1 levels, a renal biopsy would be strongly recommended. However further research necessary in order to confirm the normal range for serum Gd-IgA1.

In addition to lectin-based assays, mass spectrometry (MS), including matrix-assisted laser desorption ionization time-of-flight MS (MALDI-TOFMS) and liquid chromatography/electro- spray ionization/MS (LC/ESI/MS) have been used to detect Gd-IgA1 and to elucidate the O-glycosylation patterns of IgA1 molecules[33–35]. However, these techniques are not suitable for clinical application due to complicated sample preparation[14]. Xu *et al*. [36] analyzed Gd-IgA1 using elderberry bark lectin (SNA), peanut agglutinin (PNA) and *Vicia villosa* lectin (VVL), which recognize the α2,6-linked neuraminic acid (NeuAC), galactose (Gal) and GalNAc residues, respectively. Artocarpus heterophyllus, Glycine max and Sambucus nigra lectins have also been used to bind specific IgA1 O-glycan with similar results[37]. Moore et al[38] found that lectins from *Helix aspersa* (HAA) and *Helix pomatia* bound exclusively to IgA1 containing Gal-deficient O-linked glycans, while VVL recognized not only GalNAc in *O*-glycans, but N-linked glycans as well. Sachiko et al preferred HAA to VVL lectin for detection of Gd-IgA1[15]. Since the results were similar between the HAA and VVL groups, we recommend using the most available way to detect Gd-IgA1 in clinical practice. However, a standard method for detecting serum Gd-IgA1 should be identified and agreed upon in the future.

Moldoveanu et al found no correlation between HAA–IgA binding levels and glomerular filtration rate, histological abnormality or amount of urinary protein in Caucasian adults with IgAN [4]. Sachiko et al suggested that the level of HAA–IgA binding in Japanese adults with IgAN did not correlate with disease severity or combined therapy[15]. Furthermore, a recently described association between the magnitude of proteinuria and percent serum Gd-IgA1/IgA was not confirmed in a cohort of pediatric patients with IgAN[39]. In contrast, Camilla et al[40], examined adults and children with IgA and found a marginally significant correlation between percent Gd-IgA1/IgA and contemporaneous urinary protein/creatinine ratio and Zhao et al found that elevated Gd-IgA1 levels in Chinese patients with IgAN may affect disease progression[6].

In the current study, no significant differences were found when comparing the changes in clinical manifestations and pathology in cases of mild and severe IgAN. Among these, 4 comparisons were based on histopathologic grading: mild mesangial proliferative IgAN versus focal proliferative sclerosis IgAN; I+II versus IV+V; I-III versus IV-V, while the other one was based on absolute renal risk (ARR): 1 versus 3. Excluding the latter, the results of the meta-analysis remained the same (Fig 6). We suspect that serum Gd-IgA1 may be a specific marker for IgAN diagnosis, but not a sensitive marker to for determining the severity of disease. Since the raw data was not available in some studies, further comparisons of multi-center studies and large samples are needed.

The Gd-IgA1 level in IgAN patients was not significantly higher compared to the level in HSPN patients in either adults or children. The level of serum Gd-IgA1 was also been found to be elevated in patients with HSPN, regardless of ethnicity or age, compared with healthy controls. This is consistent with prior reports of detectable glycosylation defects in patients with HSPN[30,41] and strongly supports the hypothesis that IgAN and HSPN represent clinical phenotypes that share a common pathogenic mechanism. Krzysztof et al demonstrated that serum Gd-IgA1 levels are highly inherited in cases of pediatric HSPN[13]. The serum Gd-IgA1 level may constitute a useful tool for screening and stratification of pediatric patients at risk for HSPN as well as IgAN.

Interestingly, our analysis showed that the level of Gd-IgA1 in IgAN was significantly higher compared with other renal diseases, including minimal change disease (MCD), membranous nephropathy, biopsy-proven nephro-arteriolosclerosis, Alport syndrome, thin basement nephropathy and mesangial proliferative glomerulonephritis. Moreover, there was no significant difference in the Gd-IgA1 levels of patients with other renal diseases compared with healthy controls, suggesting that a highly elevated Gd-IgA1 level is specific to IgA nephropathy and could be used to distinguish IgAN from membrane nephropathy, MCD, Alport syndrome or other renal diseases.

Surprisingly, 2 studies indicated that serum levels of Gd-IgA1 did not differ between the IgAN patients and their first-degree relatives, suggesting the heritability of serum Gd-IgA1 levels in IgAN. Familial forms of IgAN have been reported worldwide, including in sibling pairs, families and extended pedigrees belonging to geographically isolated populations. A recent genome-wide association study (GWAS) identified multiple susceptibility loci coding for genes involved in critical mechanisms for the development of IgAN[42,43]. Identification of genes and pathways responsible for Gd-IgA1 and illness onset may ultimately lead to the development of novel therapeutic and prophylactic approaches.

### Limitations

**Fig 7:**
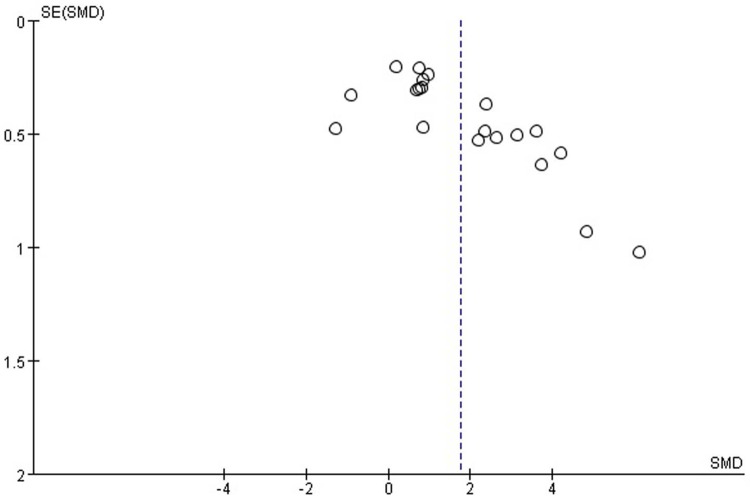
The funnel plot of the meta-analysis.

The major limitation in this study was that most included researches were published studies, which could introduce reporting bias. This is not evident from a visual inspection of the funnel plot (Fig 7), which was symmetrical. For comparisons using standard MD, the results are for reference. Regrettably, some papers were excluded due to a lack of raw data.

## Conclusion

The level of Gd-IgA1 in the serum or supernatant of cultured cells from the peripheral blood or tonsil is likely to be a useful biomarker for the diagnosis of IgAN, though the Gd-IgA1 level does not appear to be associated with disease severity. Further research is needed in order to determine the Gd-IgA1 level that indicates actual disease.

## Supporting Information

S1 Fig Pubmed search strategy of the systematic review. (JPG)

S2 Fig The forest plots of the comparation between IgAN group and control group. (JPG)

S1 File PRISMA checklist. (DOC)
