# Supplementary figures and images for: Aberrant IgA1 Glycosylation in IgA Nephropathy: A Systematic Review
Source: PLoS One. 2016 Nov 21;11(11):e0166700. doi: 10.1371/journal.pone.0166700 (PMC5117702; doi:10.1371/journal.pone.0166700)

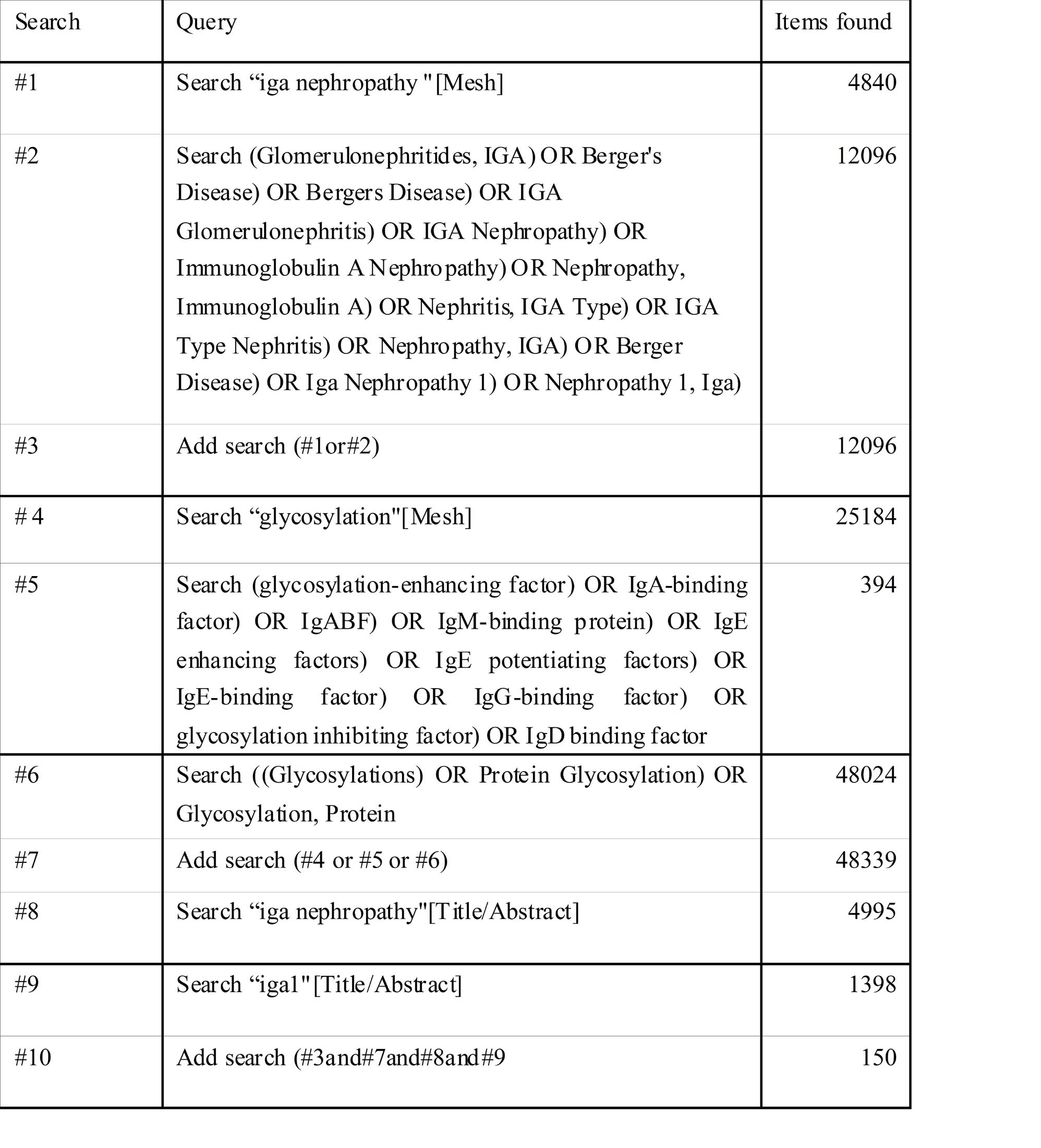

Supplement: S1 Fig — (JPG) [file pone.0166700.s001.jpg]

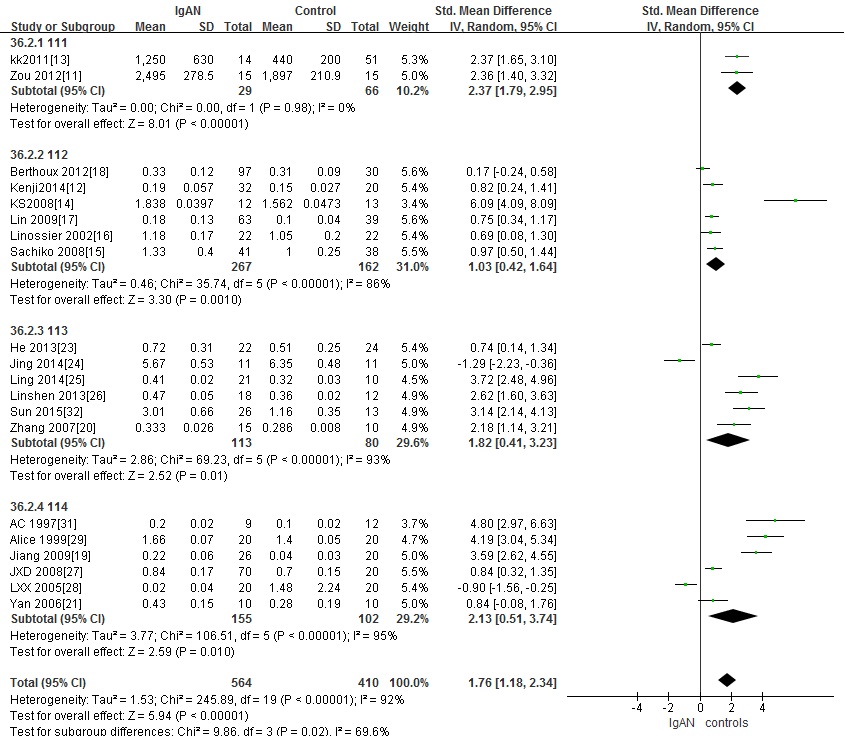

Supplement: S2 Fig — (JPG) [file pone.0166700.s002.jpg]
